# Spontaneous and in vitro fertilization pregnancies have comparable first trimester screening profiles for Down syndrome

**DOI:** 10.4274/jtgga.galenos.2018.2017.0133

**Published:** 2019-05-28

**Authors:** Yılmaz Güzel, Engin Türkgeldi, Hande Yağmur, Zeki Salar, Başak Balaban, Bülent Urman, Özgür Öktem

**Affiliations:** 1 Assisted Reproduction Unit, American Hospital, Women’s Health Center, İstanbul, Turkey; 2 Department of Obstetrics and Gynecology, Koç University Faculty of Medicine, İstanbul, Turkey

**Keywords:** First trimester screening, pregnancy-associated plasma protein-A, beta-human chorionic gonadotrophin, nuchal translucency, pregnancy, in vitro fertilization, intracytoplasmic sperm injection

## Abstract

**Objective:**

We aimed to compare the first trimester screening profiles of spontaneous (n=972) and in in vitro fertilization (IVF) pregnancies (n=339) in a population of patients who had uncomplicated singleton pregnancies comparable for maternal age, gestation, body mass index, and ethnicity.

**Material and Methods:**

A non-interventional analysis of retrospective cohort data and review of the literature.

**Results:**

All IVF pregnancies were achieved via intracytoplasmic sperm injection using the same ovarian stimulation protocol with recombinant follicle-stimulating hormone and a gonadotropin-releasing hormone antagonist, cetrorelix acetate. The means of the multiple of median (MoM) of pregnancy-associated plasma protein-A (PAPP-A) were slightly lower in the fresh (1.19±0.6 vs 1.33±0.7, respectively; p=0.056) and frozen embryo transfer (1.03±0.5 vs 1.33±0.7, respectively; p=0.036) IVF pregnancies compared with natural conceptions. However, when the medians of the MoMs of PAPP-A and beta-human chorionic gonadotrophin (β-hCG), and their distributions were compared across the mode of conception, there were no differences between IVF pregnancies spontaneous pregnancies. Furthermore, the scatterplot diagram and curve fitting regression analyses revealed no difference in the temporal relations of β-hCG and PAPP-A with each other and gestational age between spontaneous and IVF pregnancies.

**Conclusion:**

These results support the notion that uncomplicated singleton IVF pregnancies have similar first trimester screening profiles to spontaneous onceptions.

## Introduction

Prenatal screening for chromosomal abnormalities using maternal serum and sonographic markers has been integrated into routine antenatal care in many countries. First trimester screening test combines maternal age, nuchal translucency (NT), maternal serum pregnancy-associated plasma protein-A (PAPP-A), and free beta-human chorionic gonadotrophin (β-hCG) to generate a risk assessment for Down syndrome and other trisomies. This method has been reported to identify about 90% of cases of trisomy 21 with a 5% false-positive rate (1,2). An increasing number of pregnancies are achieved through assisted reproductive technologies (ART) every year. Several studies demonstrated that pregnancies conceived through ART are associated with altered maternal levels of the biomarkers of the first trimester screening, affecting the risk assessment for Down syndrome (3,4,5,6,7,8,9,10). A decrease in the maternal serum level of PAPP-A and normal or higher levels of β-hCG in in vitro fertilization (IVF) pregnancies appear to be the most consistent findings of these studies including the result of a recent meta-analysis (3,4,5,7,11,12). On the other hand, some other reports found no differences in the levels of these biomarkers between natural and IVF pregnancies (13,14,15,16).

Apparently, the inconsistent results among the studies are likely to be accounted for by several confounding factors such as heterogeneous patient populations, maternal age, and adverse obstetric outcomes. Notably, the levels of these biomarkers remained to be altered in some of these studies after adjusting for these variables and excluding cases with poor obstetric events (5,6). There are also limited data regarding the comparison of IVF pregnancies after fresh vs. frozen embryo transfer (ET) cycles in terms of their effect on the parameters of the first trimester test. Therefore, we conducted this retrospective cohort study in a homogeneous population of patients who were comparable for maternal age, gestation, body mass index (BMI) and ethnicity and had uncomplicated singleton pregnancies ending in the birth of full-term neonates. Our aims were to investigate whether (1) IVF pregnancies achieved after fresh and frozen ET IVF cycles were different from natural conceptions in terms of the first trimester test results in a patient cohort comprising uncomplicated singleton pregnancies; and (2) there were any differences in these parameters between fresh and frozen ET IVF pregnancies conceived using the same ovarian stimulation protocol with recombinant follicle-stimulating hormone (FSH) and a gonadotropin-releasing hormone (GnRH) antagonist.

## Material and Methods

### Patients

In this retrospective cohort study, the first trimester screening profiles of spontaneous (n=972) and IVF pregnancies (n=339) in a population of patients who had uncomplicated singleton pregnancies comparable for maternal age, gestation, BMI, and ethnicity were analyzed. All singleton pregnancies whose first trimester screening results were available were included. Of these, only 24 were excluded because of no information on pregnancy outcome (n=10), preeclampsia (n=4), gestational diabetes (n=2), which left 1331 for further evaluation. All pregnancies were singleton, uncomplicated and resulted in full term birth (37 completed weeks of gestation) of healthy neonates in a private hospital between January 2009 and July 2014. Nine hundred seventy-two of these were spontaneous pregnancies, whereas the remaining 339 were achieved through IVF-ICSI after the transfer of fresh (n=301) and frozen (n=38) embryos. The study was approved by the institutional review board of Koç University (IRB# 2015.207.IRB2.077, date: 10.09.2015). The study was performed in accordance with the ethical standards described in Declaration of Helsinki. For each patient, age at the time of the test, ethnicity, weight, smoking status, diabetes history, any previous pregnancy with trisomy, family history of trisomy, date of last menstrual period, crown-rump length (CRL), nuchal translucency, and serum PAPP-A and β-hCG levels and their multiple of median (MoM) values were recorded. All patients had Turkish ethnic background. A systematic literature search was performed using the key words provided to retrieve the relevant articles.

### Fertility treatment

Fertility treatment records were reviewed to identify IVF-ICSI pregnancies after fresh and frozen ET. All embryos were generated via IVF-ICSI using ovarian stimulation with recombinant FSH (Gonal-F) and a GnRH antagonist, cetrorelix acetate. In fresh transfer cycles, 8% progesterone gel was started vaginally once a day on the day of the oocyte pick-up, and the frequency was increased to twice a day on the transfer day. No estrogen support was administered for fresh transfers. For frozen-thawed ET, endometrial preparation was provided with oral estrogen and vaginal progesterone gel. Patients were given a tablet of 2 mg estradiol/day in the first 4 days of the cycle, 2 tablets/day on the next 4 days, and 3 tablets/day thereafter. Eight percent progesterone gel was administered once daily 3-5 days before the transfer and twice a day starting on the day of the transfer. All medications were used until the 8^th^ gestational week if pregnancy was achieved.

### Risk assessment using the first trimester combined test

All patients underwent the first trimester screening test between 11+0 and 13+6 weeks of gestation. Gestational age was determined using CRL. NT was measured according to the Fetal Medicine Foundation protocol by the same physicians (17). For all cases, blood samples was obtained on the day of the NT scan for measurement of maternal PAPP-A and β-hCG levels and analyzed using a Delfia^©^ Express 6000 Immunoanalyzer (PerkinElmer, Waltham, Massachusetts, USA). Down syndrome risk was calculated using the LifeCycle 2.2 Rev. 4 software (PerkinElmer, Waltham, Massachusetts, USA).

### Statistical analysis

Demographic characteristics of the patients (maternal age, body weight, gestational age, BMI, and CRL) are expressed as mean ± standard deviation (SD) (Table 1). The MoM of the markers of the first trimester screening (NT, β-hCG and PAPP-A) are expressed as mean, median, SD, and percentile (25, 50, 75) (Table 2). The variables in the baseline demographic characteristics and the means of the MoMs between spontaneous and IVF (overall) pregnancies were compared using the t-test and Mann-Whitney U test, respectively. Additionally, spontaneous and IVF pregnancies conceived after fresh and frozen ET cycles were compared using the Kruskal-Wallis test and Dunn’s multiple comparison posthoc test. The variables were also tested as to whether they were distributed normally using the Kolmogorov Smirnov one-sample test. The medians of the MoMs and their distributions were compared using Wilcoxon’s signed-rank test to explore if the mode of conception had any influence on these parameters. The two-tailed pearson correlation test and linear regression analysis were conducted to investigate the relationship among the variables in the first trimester screening test. P<0.05 was considered significant. Data analysis was performed using SPSS (version 21; SPSS Inc., Chicago, IL, USA).

## Results

Baseline characteristics of the study population are shown in the Table 1. Control and IVF-ICSI patients were comparable in terms of mean age (31.5±3.7 vs 32.2±4.7 years, respectively; p>0.05), weight (64.5±10.1 vs 63.6±9.6 kg, respectively; p>0.05), BMI (20.8±2.5 vs 21.2±3.4 kg/m^2^, respectively; p>0.05), gestational age (89.1±4.7 vs 89.1±5.6 days, respectively; p>0.05), and CRL (64.1±5.7 vs 64.2±6.1 mm, respectively; p>0.05).

### Comparison of the mean MoM levels of the test biomarkers between spontaneous and IVF-ICSI pregnancies

Fetal NT and maternal blood levels of β-hCG and PAPP-A are expressed as MoMs. The mean ± SD, median, and percentiles (25, 50, 75) of the MoMs are shown in Table 2. First, we compared the means of the MoMs between spontaneous and IVF-ICSI (overall) pregnancies as two independent samples from a continuous field. There were no significant differences between these two different modes of conception in the mean MoM levels of β-hCG (1.24±0.8 vs 1.29±0.9, respectively; p>0.05), PAPP-A (1.33±0.9 vs 1.18±0.8, respectively; p>0.05), and NT (1.06±0.4 vs 1.11±0.4, respectively; p>0.05) (Table 1).

Then, IVF pregnancies were subgrouped into fresh and frozen ET cycle IVF pregnancies and a multiple comparison was made among spontaneous, fresh, and frozen IVF pregnancies. The means of the MoM of PAPP-A were significantly lower in the fresh (1.19±0.6 vs 1.33±0.7, respectively; p=0.056) and frozen ET (1.03±0.5 vs 1.33±0.7, respectively; p=0.036) IVF pregnancies compared with spontaneous pregnancies. The MoMs of β-hCG (1.24±0.6 vs 1.26±0.8 vs 1.48±0.8, respectively; p>0.05) and NT (1.06±0.8 vs 1.13±0.8 vs 1.01±0.8, respectively; p>0.05) showed significant variations among spontaneous, fresh, and frozen ET IVF pregnancies. Furthermore, IVF pregnancies occurring after fresh ET cycles were not different from those after frozen ET cycles in terms of the mean MoM levels of these biomarkers (Table 2).

### Comparison of the medians of the MoMs across the mode of conception and their distribution between spontaneous and IVF pregnancies

When the medians of the MoMs of β-hCG, PAPP-A and NT of IVF pregnancies were compared with the corresponding medians in the spontaneous pregnancies, no significant differences were found between spontaneous and IVF pregnancies regarding the distribution and median MoMs of the biomarkers (Table 2). The asymptotic significances were as follows: 0.544 for β-hCG, 0.89 for PAPP-A, and 0.53 for NT (Table 2). The MoMs of β-hCG, PAPP-A, and NT were not normally distributed in either spontaneous or IVF pregnancies on the Kolmogorov-Smirnov test.

### Comparison of spontaneous and IVF pregnancies for the temporal relationship among β-hCG, PAPP-A, and gestational age

**Figure 1:**
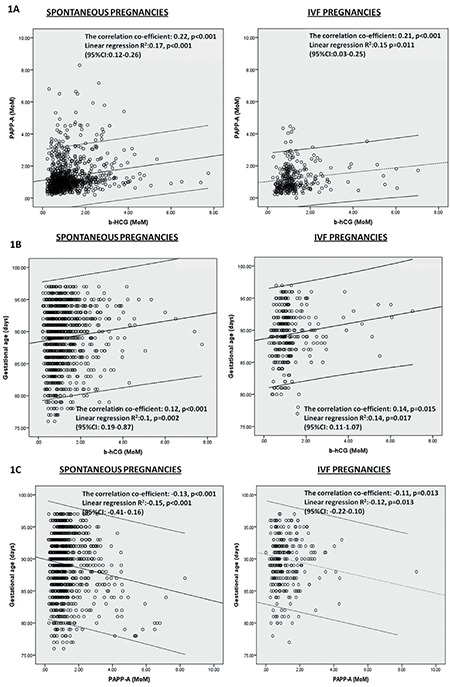
Temporal relationship between gestational age, β-hCG and PAPP-A in spontaneous and IVF pregnancies β-hCG: β-human chorionic gonadotropin, PAPP-A: Pregnancy associated plasma protein-A , IVF: In vitro fertilization

Both β-hCG and PAPP-A are produced by trophoblastic tissue during pregnancy (1). Thus, we investigated the temporal relationship between these biomarkers in spontaneous and IVF pregnancies using correlation analyses. Two-tailed pearson correlation analysis revealed that β-hCG was positively correlated with PAPP-A in both spontaneous (correlation co-efficient: 0.22, p<0.001) and IVF pregnancies (correlation co-efficient: 0.21, p<0.001). There was also a positive correlation between gestational age and β-hCG in spontaneous (correlation co-efficient: 0.12, p<0.001) and IVF pregnancies (correlation co-efficient: 0.14, p=0.014). Gestational age was inversely correlated with PAPP-A in spontaneous (correlation co-efficient: -0.13, p<0.001) and IVF pregnancies (correlation co-efficient: -0.11, p=0.013). In the linear regression analysis, β-hCG (R^2^=0.10, p=0.002) and PAPP-A (R^2^=-0.15, p<0.001) remained significantly associated with gestational age in spontaneous conceptions. Similar associations were found between β-hCG and gestational age (R^2^=0.14, p=0.017), and between PAPP-A and gestational age (R^2^=-0.12, p=0.013) in IVF pregnancies (Figure 1).

## Discussion

We used different statistical models in this study to analyze and compare the first trimester screening test results of spontaneous and IVF pregnancies. Similar to some previous reports, we showed that IVF pregnancies had slightly lower PAPP-A, and similar β-hCG and NT levels compared with spontaneously conceived pregnancies when both groups consist of uncomplicated singleton pregnancies comparable for maternal age, gestation, weight, BMI, and ethnicity. Also, we find no differences in these parameters between the pregnancies after fresh and frozen ET cycles.

A number of studies examined the possible effects of IVF on first trimester screening test results. These studies are summarized in Table 3. The majority of them reported decreased MoM levels of PAPP-A for both IVF and ICSI pregnancies (3,4,5,6,7,8,9,10) as the most consistent finding (Table 3). As a result of lower PAPP-A levels, higher false-positive rates were reported in the combined screening test of IVF pregnancies (18). PAPP-A is a placental derived protein and its synthesis is defective in Down syndrome due to the impaired differentiation of cyto to syncytiotrophoblasts in the placenta (19). Therefore, maternal PAPP-A levels are lower in fetuses with trisomy 21. However, given that the incidence of births of the fetuses with trisomy 21 was not increased in IVF pregnancies, there must be some other mechanisms that could provide a plausible explanation for the observed decrease in the PAPP-A level and higher false-positive rates of the first trimester screening in these pregnancies. To date, several hypotheses have been put forth to explain this phenomenon. Tul and Novak-Antolic (10) demonstrated that there was an inverse association between the number of aspirated oocytes and PAPP-A MoM values and that inhibin A, a product of the corpus luteum, was increased with decreasing PAPP-A and increasing the number of oocytes retrieved. Based on these findings, the investigators hypothesized that ovarian stimulation was associated with the generation of multiple corpora lutea and higher endogenous levels of inhibin A, which in turns inhibits the secretion of PAPP-A. Currently this hypothesis lacks biologic validation and cannot explain the others’ findings showing normal levels of PAPP-A in IVF pregnancies in comparison to controls (13,14,15,16). Furthermore, lower maternal PAPP-A levels were also reported in IVF pregnancies after frozen ET cycles, in which the ovaries are not stimulated, and therefore there are no multiple corpora lutea or elevated serum levels of inhibin-A (3,20).

Decreased PAPP-A levels are not specific for Down syndrome because it is also measured at lower levels in euploid pregnancies complicated by defective placentation such as pre-eclampsia and fetal growth restriction (21). This fact raises a question as to whether lower PAPP-A levels may indicate impairment of early implantations of the pregnancies in IVF populations. Currently, there is no good evidence to prove this claim, but it is known that IVF/ICSI pregnancies are more prone to developing adverse obstetric outcomes than natural conceptions. Thus, could lower PAPP-A levels in these pregnancies be the harbinger of poor obstetric events in the future? The answer is probably no because maternal PAPP-A level continues to remain low even in uncomplicated IVF pregnancies when all cases with obstetric complications were excluded (5,6). Furthermore a recent study comparing first trimester trophoblast volume and placental bed vascular volume between IVF/ICSI (n=70) and normal singleton pregnancies (n=84) using a virtual organ computer-aided analysis system demonstrated no differences in these parameters between spontaneous and IVF pregnancies (22).

Interestingly, it was shown that subfertility itself and the etiology of infertility may also alter the levels of maternal PAPP-A. For instance, time to pregnancy (TTP) is a clinical tool to assess uterine receptivity/subfertility. Ranta et al. (23) demonstrated that the median/geometric mean multiple of MoM of PAPP-A was significantly lower (p<0.01) in women with a TTP over 25 months (0.89/0.83 MoM) and in the IVF group (0.95/0.84 MoM) compared with the reference group (1.01/1.03 MoM). However, first trimester β-hCG and NT MoMs were not statistically different between the study groups. Consequently, the proportion of the test screening positives was significantly higher in women with TTP ≥25 months (12.9 vs 2.1%), but not in the IVF group (2.6%). Regarding the effect of infertility on the test results, a record-linkage study showed that PAPP-A levels were reduced when the infertility was reported to be of female-only etiology (0.82 MoM), male-only etiology (0.85 MoM), and when a combination of male and female etiologies were present in the couple (0.82 MoM) (3).

Advanced maternal age among patients who become pregnant after IVF/ICSI appears to be another factor responsible, at least in part, for the higher false-positive rate of the test in these patients. However, when age-matched controls were used and age-adjusted analysis was perfomed, the false-positive rate remained persistently high in these IVF pregnancies (3,24). Of particular note, clinically recognized twin pregnancies can be spontaneously reduced to singleton in IVF/ICSI twin pregnancies known as vanishing twin phenomenon. These pregnancies are characterized by higher maternal MoM values of PAPP-A and β-hCG (25,26). Therefore, the results should be carefully analyzed when there is another gestational sac empty or filled with a dead fetus. PAPP-A is a protease of insulin-like growth factor (IGF) binding protein-4 produced by decidua and trophoblastic tissue, and plays critical roles during human implantation such as the regulation of IGF bioavailability in the placental bed (27,28). Therefore, taken together, these findings suggest that IVF pregnancies are likely to be different from spontaneous pregnancies and that IVF treatment itself can modify the implantation process leading to lower PAPP-A levels, which are not always associated with clinically recognized abnormal pregnancy outcomes.

PAPP-A circulates at very low levels in non-pregnant women. Like β-hCG, it is produced at high levels by the placenta during pregnancy (29). We could not find any differences in the temporal relation of β-hCG and PAPP-A with each other and gestational age between natural and IVF conceptions. This situation provides supporting evidence for the notion that IVF pregnancies are not different from spontaneous pregnancies in terms of the first trimester biomarkers.

The other biochemical marker in the first trimester screening test is β-hCG. High levels of β-hCG in the first trimester are associated with increased risk of Down syndrome, whereas its elevated levels in the second trimester are more related to poor obstetric outcomes (30). Although some studies reported increased (5,11,14,15,31) or decreased β-hCG MoM levels in ART pregnancies compared with controls (6), a great majority of the studies including ours found no difference in the levels of β-hCG levels between natural and ART pregnancies (3,4,6,7,9,10,16,20,32). These inconsistent findings have been attributed to small sample sizes, the heterogeneity of study populations, and the differences of β-hCG levels at different gestational weeks (3,18,32).

Another important finding of our study is that IVF pregnancies after fresh ET cycles had similar first trimester screening profiles to those of frozen ET cycles. This information could be relevant because a limited number of studies have thus far analyzed the first trimester screening profiles of ART pregnancies conceived after fresh and frozen ET cycles and reported varying results (3,4,7,9,20). Some of these studies reported significantly reduced median PAPP-A MOM values for pregnancies after fresh IVF and ICSI cycles, whereas median PAPP-A MOM values of pregnancies after frozen cycles were similar to those of spontaneous pregnancies (4,7). Hui et al. (20) studied both IVF and ICSI cycles with fresh and frozen-thawed ET. PAPP-A was significantly decreased in fresh IVF, fresh ICSI, and frozen ICSI pregnancies but not in frozen IVF pregnancies. Fresh and frozen ICSI groups had comparable median PAPP-A MoMs (20). In another study, Matilainen et al. (9) compared spontaneous pregnancies, fresh IVF/ICSI cycles, and frozen-thawed ET with and without hormone stimulation. The median PAPP-A MoM value for fresh cycles was significantly lower than in the control group. Frozen transfer groups, with and without hormone treatment, had lower MoM values than the control group. Interestingly, fresh and frozen-thawed transfer cycles in which exogenous hormones (follicle-stimulating agents, or any combination of estrogen and progesterone) were used had significantly lower PAPP-A values when compared with fresh and frozen-thawed cycles without hormones as shown by Amor et al. (3) who compared 773 fresh cycles with 573 frozen-thawed cycles. A recent meta-analysis documented that free β-hCG tests showed slightly higher values in the ICSI group than controls (RR=1.09, 95% CI: 1.03-1.16) but not in the IVF group (RR=1.03, 95% CI: 0.94-1.12). Pregnancy-associated plasma protein-A values for IVF/ICSI, IVF, and ICSI showed lower values in comparison with controls (RR, 95% CI: 0.85, 0.80-0.90; 0.82, 0.74-0.89 and 0.83, 0.79-0.86, respectively). The nuchal translucency measurement showed no statistical differences between study groups (IVF and ICSI) and controls (RR=1.00, 95% CI: 0.94-1.08 and RR=1.01, 95% CI: 0.97-1.05, respectively) (12).

NT is the only ultrasound marker in the first trimester combined screening test. Most studies have shown similar mean MoM values for NT in natural and IVF pregnancies (3,5,6,9,10,13,16,24). A few studies found thicker (33) and thinner NT values (7,32) for the general IVF population. However, all studies on fresh and frozen-thawed ET cycles reported comparable NT MoM values for both. In support of this, our results revealed no significant variations in the MoMs of NT between spontaneous and IVF pregnancies.

Our results show that the results of the first trimester combined test did not differ between natural and IVF pregnancies when a homogenous patient population comparable for maternal age, gestation, BMI, ethnicity, and ovarian stimulation protocol was analyzed. On the other hand, test results should be interpreted cautiously in IVF pregnancies because many studies reported that the biochemical markers of the test might be affected by several factors in these pregnancies such as the mode of conception, etiology of infertility, maternal age, ovarian stimulation, and vanishing twins.

## Figures and Tables

**Table 1:**
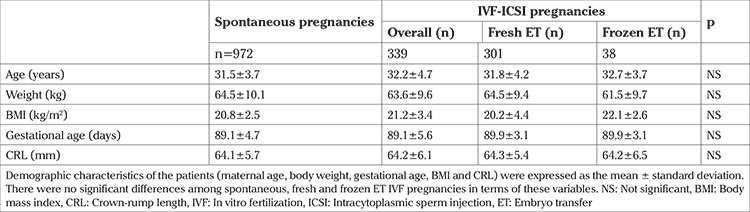
Comparison of the demographic characteristics of the spontaneous and IVF pregnancies

**Table 2:**
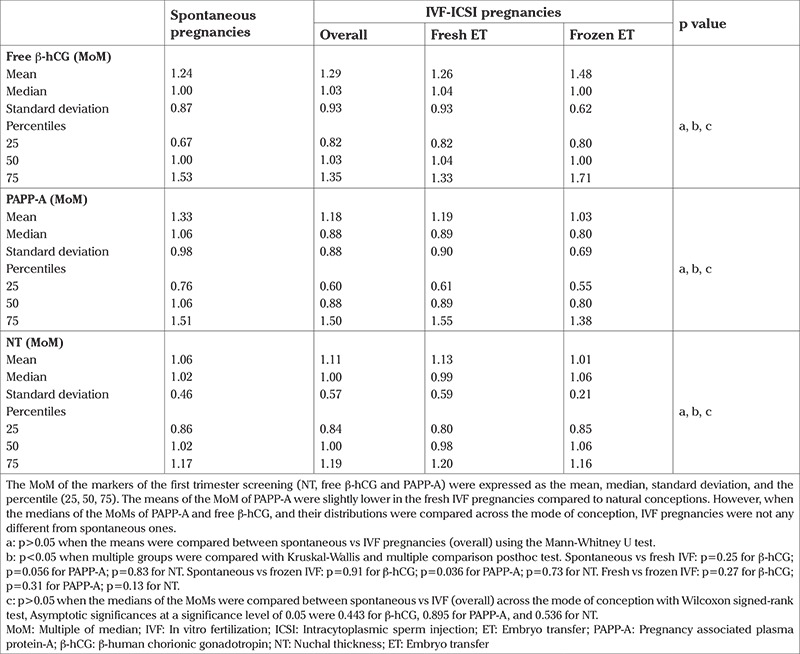
Comparison of the first trimester biomarkers among spontaneous, fresh and frozen cycle IVF pregnancies

**Table 3:**
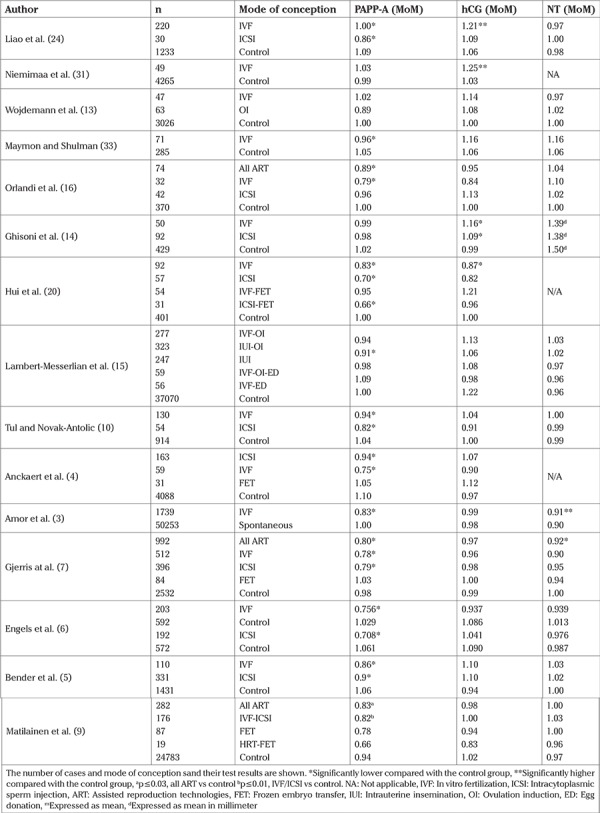
Summary of the findings of the previous studies evaluating PAPP-A, b-hCG and NT MoM values in spontaneous and IVF pregnancies
